# Human Life as *Terra Nullius*: Socially Blind Engineering in Facebook’s Foundational Technologies

**DOI:** 10.1007/s13347-025-00971-9

**Published:** 2025-10-14

**Authors:** João C. Magalhães, Nick Couldry

**Affiliations:** 1 School of Arts, Languages and Cultures, Centre for Digital Trust and Society, University of Manchester, Oxford Rd, Manchester, M13 9PL UK; 2 Department of Media and Communications, London School of Economics and Political Science, Houghton Street, London, WC2A 2AE UK

**Keywords:** Social engineering, Social media platforms, Facebook, Data colonialism, Banality

## Abstract

Critical platform scholars have long suggested, if indirectly, that social media power is somehow akin to social engineering. This article argues that the parallel is analytically productive, but for reasons that are more complex than has previously been appreciated. By examining Facebook’s foundational technologies, as described in patents that sought to protect the company’s early innovations, we argue that, unlike previous technocratic attempts to reconstruct society, the platform’s equally consequential rendering of social reality into a legible and controllable social graph involved no substantive vision of the social world at all. Rather, the company engaged in a form of *socially blind engineering*, misrecognizing the actual social world as a *terra nullius*, as if it had no inhabitants who needed to be taken into account, and so was a domain from which profit could be extracted with relative impunity. In so doing, we develop a conceptual vocabulary to understand the widely-criticised recklessness that, notwithstanding some more charitable recent readings, marked the early Facebook – and that might still influence the tech sector as a whole.

## Introduction

Few doubt that social media have had major consequences almost everywhere for the social world and indeed for the politics that is rooted in the social world. Although debates are sometimes diverted by the more diffuse question of our use of mobile phones (Haidt, 2024), the arguments that these platforms^1^ have at the very least amplified (though not necessarily caused) threats to social life such as polarisation and declining mental health are now being taken seriously as a starting-point for government policy and public debate (for a summary of the debate, see Couldry, 2024). Underpinning these concerns is the realisation that, as they became central global spaces of interaction, for-profit platforms have enabled the disruption, erosion, and warping of key social values and processes, a form of symbolic violence that co-exists with any eventual expansion of self-expression, entertainment, and convenience these digital spaces enabled (Couldry & Mejias, 2019; Magalhães & Yu, 2022).

Explaining the relationship between this vast, historic reshaping of the social world (i.e. the constructed reality that emerges through our embedded interactions and related understandings, as Berger and Luckmann (1966) understand it), and the digital materiality of social media has been one of the central objects of critical platform studies. This literature has long suggested, if indirectly, that, in producing, designing, and programming sociality, platform power is comparable to social engineering (see e.g. Zuboff, 2019: 216 on “behavioural modification”; cf. van Dijck, 2013: 3–23, 178 on “engineering sociality”) – all terms closely associated with high modern state projects that strove to make society fully knowable and actionable (Scott, 1998). To think of platforms as the heirs of earlier social engineers illuminates how acutely centralised and highly technical software has come to shape human life at a planetary scale. But it also forces us to question whether Silicon Valley entrepreneurs, capitalists, and computer scientists, in designing and enhancing their code-and-software-based products, have been engaged in a comparable project to that of the statesmen, public officials, and activists who built cities and institutions to bureaucratize modernity between the 18th and 20th centuries.

This article argues that the parallel between platforms and social engineering is analytically productive, but for reasons considerably more complex than has previously been appreciated. Analysing 133 patents that detail what we name Facebook’s foundational technologies, the article suggests that, while previous attempts to reconstruct society were driven by grand and morally-charged planning, Facebook’s rendering of social reality into a legible and controllable “social graph” involved *no substantive vision of the social world at all*; instead, it was characterized by a form of *socially blind engineering* that is constitutive of the power the platform wielded and crucial to understanding the mechanisms behind its consequences. Proposing that platform designers ought to at the very least recognize users as moral subjects (Honneth, 2014) and aligning itself with the wider thesis of data colonialism (Mejias & Couldry, 2024), the article argues that what made this process possible was a generalised understanding of human life itself as *terra nullius*, that is, a space of free exploitation (for platforms as a sort of *terra nullius*, see Cohen, 2019: 50; Couldry & Mejias, 2019: 9). Similarly to historical colonizers, Facebook treated the social world as a no one’s land that could be simply taken and used without consequences – a large-scale *mis*recognition of the actual social world. In so doing, we develop a conceptual vocabulary to discuss claims that the platform was invented by “careless people” who barely understood “what power has bought and brought them”, as Facebook former executive Sarah Wynn-Williams writes (2025: 10). Her memoir, which built on earlier revelations by Frances Haugen and Sophie Zhang and triggered a strong public reaction, offers personal evidence confirming our findings. But, by analysing the early patents which enacted this misrecognition of the actual social world by constructing a shadow social world over which Facebook retained absolute control, the article shows practically how such recklessness was performed and sustained, and the key design mechanisms it used. The result is to explain how even the strongest critiques to date of the company now known as Meta fall short of the reality.

Our argument unfolds as follows. After exploring the associations between social engineering and social media, we explain the article’s case study (the foundational systems and designs that paved Facebook’s rise and consolidation, between 2004 and 2011), and how we analysed these technologies through their patents. Our qualitative analysis of these documents reveals that the engineering of Facebook depended on three assumptions, all of which were useful to the platform’s business model: that basic social definitions, such as what counts as an “actor” and a “relation”, can be entirely plastic and manipulable; that norms can be translated into probabilistic mechanisms, whereby behavioural metrics seemingly replace normative evaluation; and that the process of sociality itself can be turned into a potentially deceptive simulation tool, whose most important drivers become hidden from users. Together, these assumptions compose a ruthlessly extractivist approach to the social – where social ontology, normativity, and practices are understood as mere shells of the reality they refer to, computational models that can be synthetically reproduced and then harvested solely for Facebook’s profit.

Through careful consideration of the early patents which built the parallel, data-extracting world in which we were lured to act, while prima facie just performing natural social interactions, we show that this misrecognition was neither accidental nor a version of ‘good intentions gone wrong’ explanation of high modern social engineering catastrophes (Scott, 1998). This is all the more important, when versions of this explanation have recently resurfaced in commentary on social media. For example, Nicholas Carr’s argument (Carr, 2025) that it was Facebook’s failure to grasp the consequences of removing normal boundaries between communication situations that converted Zuckerberg’s original communicative idealism into a social problem; or (more critically) Marion Fourcade and Kieran Healy’s argument that Facebook merely “accommodated and then egged on people’s social and creative impulses.. while also converting that tendency into profit” (Fourcade & Healy, 2024: 64), as if Facebook’s vast project were merely happy opportunism. The clear architectural intent of the early patents flatly contradicts these more generous accounts. At the same time our argument is that, because the terra nullius Facebook sought to manage was not land but a “data territory” (Mejias & Couldry, 2024: Chap. 2), created through code and installed into the social world through what Wendy Chun calls the “executive” power of code (Chun, 2011: 27), code protected by specifications in the patents examined, its mechanism represents not explicit social engineering so much as banal bureaucratic power (Arendt, 1963). But this banality of process did not mean the social *effects* were banal, as extensive public debate in the past decade has recognised. As a result, it is Facebook’s *disregard* for the social consequences of its blind social engineering – its refusal to acknowledge its’ platform’s users as real moral subjects – that is most shocking.

## Social Media and Social Engineering

The term “social engineering” designates large-scale projects whereby technocratic elites develop top-down epistemological and political techniques to render the world below them legible and controllable, creating “a high probability” that people – technocrats’ “raw material” – will act according to certain “visions” of what a good society ought to be (Podgorecki et al., 1996: 1). In their most radical embodiments, these projects aimed at manufacturing new social identities that could justify the violent exclusion of particular “others” (Browning & Siegelbaum, 2009: 231). Others argued that, as long as it followed the “piecemeal” approach of “proper” social science or was guided by genuinely democratic intentions, as illustrated by the Swedish welfare state, social engineering could bring about unprecedented moral progress (Popper, 1957; Etzemüller, 2014).

Somewhere between these two poles lies the thesis that “high modern” state bureaucracy in the 19th and 20th centuries engendered several “well-intended schemes to improve the human condition” that went wrong, often catastrophically, in James Scott’s formulation (1998: 4). Ranging from city building in Brazil to communist revolution in Russia, these initiatives enacted a “totalising”, “sweeping vision of how the benefits of technical and scientific progress might be applied.. in *every* [emphasis added] field of human activity” (Scott, 1998: 90), generating new “habits, work, living patterns, moral conduct, and worldview” (Scott, 1998: 89). High modernity’s failures (millions of “lives lost.. and disrupted”) can be traced back to the chasm between elites’ lofty goals of radical improvement and the social reality their ideas were designed to impact (Scott, 1998: 3). In other words, these initiatives hinged on the assumption that society was deserving of improvement but undeserving of actively participating in such improvement, which made possible the implementation of “utopian plans” with “an authoritarian disregard for the values, desires, and objections” of those who were expected to benefit from such projects (Scott, 1998: 7). The great novelistic dystopias of the 20th century (Huxley’s *Brave New World* and Orwell’s *1984*) satirized that vision.

Scholars have often associated, if indirectly, high modern social engineering and platform power (Zuboff, 2019; Fourcade & Healy, 2017; Frischmann & Selinger, 2018). The comparison is warranted. Like high modern states, social media are highly centralised and technocratic organizations that affect a large number of people by translating social life into a fully legible and controllable bureaucratic language – digital data. As computational artifacts, social media can be defined as incessantly engineering *social graphs*.^2^ By this, we mean the databases and mathematical models whereby platforms store and process the real-world information they acquire through pervasive surveillance: not only on users but also objects, places, organizations, and the ways these things and people are linked (Alaimo & Kallinikos, 2017: 175; van Dijck, 2013). Reliant on seemingly “objective” homophilic techniques that are in fact rooted in earlier projects of genetic redesign (Chun, 2021; cf. Chan, 2024), these sprawling algorithmic systems materialise a form of “engineered” sociality that renders people’s relations and activities “formal, manageable, and manipulable” (van Dijck, 2013: 12). As a consequence, “[h]ow users are related to one another occurs against a platform context that is ceaselessly ploughed and reordered by similarity and popularity scores” (Alaimo & Kallinikos, 2017: 185). Engineering ever more profitable social graphs is the reason why companies constantly attempt to nudge, coerce, and reinforce specific actions, such as signing up for their services, generating data about themselves through interaction, and clicking on advertisements (Zuboff, 2019: 536–542). If it is rare for critical scholars to equate platform power with social engineering unproblematically, they nonetheless often imply that, like the latter, the former is driven by a normative core. As “computational means of assembling and organizing,” social media’s “programmed sociality” always “already embody certain norms and values about the social world” (Bucher, 2018: 4).

It is analytically useful, however, to unpack the immense *differences* in the object and scope of these two forms of engineering. High modern states’ “raw material” was the messy physical reality of actual societies, which entailed a colossal friction between legibility and controllability. Even the most accurate representation of the world could not, of itself, automatically transform the world – bureaucratic modelling needed to be socially enforced. With social graphs, the differences between the map and the territory become harder to determine. People after all always in some form interact socially. By enticing users to perform ordinary social actions on their platforms, platform owners could take advantage of physical actions that in real life are quite limited – e.g. looking at and touching on screens –with their reasons and meanings being ultimately irrelevant to the goal of profit extraction. Focused on building algorithmic worlds from which economic value can be directly extracted by the generation of attention, social media companies do not primarily care *why* users act as they do – as long as their datafied actions are aligned with the companies’ corporate objectives. In this sense, these companies *do not* see ‘like’ states, to use Scott’s language: for the “reality” they aim to engineer is not social territory, but databases reached via dashboards and coding interfaces.

That platforms maintain such a narrow relationship with the actual social world is expected. Conceived as mere products, these systems lack high modern states’ totalising goals, despite the discourse their controllers have eventually adopted (more on this shortly). Rather than generating a new social order, companies merely focused on designing and improving their social graphs. On top of those social graphs, social media construct larger platform ecologies in which users participate, that together operationalise the purposes of the social graph, from moment to moment, as real social actors interact with it in what platforms call “run time”.

And yet, social media did end up transforming society, to the surprise of their own inventors (Karppi & Nieborg, 2020). The stark disconnection between platforms’ relatively narrow objectives (to design profitable social graphs) and the destructive consequences associated with that strategy (such as the rise of authoritarian populism, declining social trust, the decay of institutional authority, such as that of science and professional journalism) comprise what we call the indeterminacy of platform power.

This indeterminacy has been neglected in the critical literature about social media which either assumes in these companies a normative drive to engineer the social world (as explained above) or invokes a new version of Scott’s (1998) theory about social engineering’s failures: “a story of the hubris of good intentions” (Vaidhyanathan, 2018: 3). This latter idea has been echoed by multiple former employees of Facebook as well, one of whom said that “it is very common” for “humans to develop things with the best of intentions and for them to have unintended, negative consequences” (Karppi & Nieborg, 2020: 2639).

Platforms have certainly tried to define themselves as driven by utopian intentions. But in the context of our analysis below, companies’ and their leaders’ statements about themselves appear to be more post-factum attempts to explain the indeterminacy of their power than evidence of social planning. In a representative example, one year after creating Facebook, Mark Zuckerberg still described it as a simple “utility” for university students; it was only in 2010 that his ideas about “the social” seemingly congealed “into a more explicit” normative vision: that the network’s mission was “giving people the *power* to share and *making the world* [added emphases] more open and connected” (Hoffman et al., 2018: 204–205). Not that the assumptions of impartiality entailed in the initial talk about “utility” disappeared. Facebook and other platforms have long tried to straddle two ideas: that their inventions actively enforce freedom and equality (Haupt, 2021; Rider & Murakami Wood, 2018) while remaining neutral and thus shielded from accountability (Gillespie, 2010). The semantic untenability of this discourse is revealing of its ideological function. This is not to say that platform designers and employees, as individuals, did not come to envision their work as having a social impact. Their very decision to concoct these justifications suggests they were unequivocally aware of (even if not directing) their products’ wider societal implications. Nor are we saying that they necessarily lied when they described these implications as socially positive. Yet even if they have come to personally believe in this narrative, there is no convincing evidence that platforms, as organizations, were designed to promote any well-defined utopia.^3^

That the inventors of social media do not appear to be primarily motivated by the same dreams of social improvement of high modern utopians does not mean that Silicon Valley engineers could – or should – ignore the moral consequences of their creations. Any technology that becomes sufficiently widespread may impact, and potentially harm, the social world – and, by extension, everyone who inhabits it. This is especially true of the for-profit digital platforms discussed in this article: systems that not only mediate human interaction directly, but were always intended to reach the broadest possible audience.

Given this, re-examining the normative grounds for critique of social media design is essential. Rather than developing a comprehensive ethical theory of platform design – a task beyond the scope of this article – we take a simpler but sufficient route by grounding our analysis in the neo-Hegelian social ethics of Axel Honneth (2014). For Honneth, the central ethical aim of modernity is individual autonomy, a value that can only be developed within *institutions of recognition*. These are shared ideas, norms, and practices through which individuals come to understand that fulfilling others’ desires is a condition for the fulfilment of their own – and to live accordingly in their everyday interactions (Honneth, 2014: 45). Institutions of recognition are thus “at once the basis and the space of realization” for what he calls social freedom (Honneth, 2014: 49). A particularly useful, if underdeveloped, aspect of Honneth’s theory for our purposes is his claim that such institutions do not arise spontaneously or randomly; rather, they must be “*designed* [our emphasis] before the subjects” (Honneth, 2014: 59).

Whether understood positively (as actively promoting mutual recognition) or negatively (as merely avoiding obstructions to mutual recognition), the existence of such institutions depends on the explicit intention of their designers to make mutual recognition socially possible. This intention, in turn, rests on what we define as the fundamental obligation of social designers: to recognize the social subjects whose lives their designs affect as moral beings, who are not only capable of but also entitled to social freedom. One might see platforms as social institutions in their own right or as shaping pre-existing ones. Either way, their designers clearly function, whether they intend it or not, as de facto social designers – and are therefore equally bound by the same obligation. This may seem like a modest expectation. Yet, as the rest of this article shows, a particular type of failure to meet this basic duty seem to have played a central role in the invention of the most paradigmatic of all social media: Facebook.

The next section outlines how we empirically explored the indeterminacy of this platform’s power beyond the discourses that have been used retrospectively to justify it.

## Studying Facebook’s Foundational Technologies

As the world’s largest platform since 2008, Facebook has played an unparalleled role in pioneering and exemplifying what networked spaces are and can be. Yet Facebook is itself too complex – some narrowing is necessary. Thus, we decided to study Facebook’s *foundational technologies*, the systems that paved the way for the company’s social and market dominance. We follow Levy, who, in the most detailed history of the company, states that the “troubles that [have] plagued [Facebook’s]” entire history are “almost all rooted in decisions” about the design of their technologies that were “made in its earlier years” – namely, between 2004 and the end of 2011, just before their Initial Public Offering in May 2012 (Levy, 2020: 38; see also Wynn-Williams, 2025: 344). On this view, there is something fundamental about Facebook to be learned from those early technologies: the kernel of a certain way of approaching the social world that structured both the company’s emergence, as well as its continuous transformations and centrality.

The specific instantiations of these early technologies might have been discontinued, but the descriptions of their underlying mechanisms have largely survived in detailed patent applications, a consequence of Facebook’s push to concentrate not only users but also intellectual property (Rikap, 2021). Patents might appear to be mere legal instruments whereby an organization claims ownership over potentially profitable inventions. Researchers, however, have long seen them as far richer documents, “fascinating sources of social and technical data” (Jungnickel, 2018: 5). Surely, patents are not manifestos in which inventors explicitly proclaim their social views. However, behind their dryly technical, painstaking descriptions of how artifacts and systems work, one can interrogate “the horizons and possibilities of technology development” within an organization or field (Magalhães & Avella, 2023, as cited in Leix Palumbo & Prey, 2024; for other critical works that examine patents, see Hlongwa & Talamayan, 2023; Delfanti & Frey, 2021; Elmer, 2019; Bucher, 2018; Rieder, 2012; Zuboff, 2019).

We used Lens, a patent search engine, to search for all US patents filed between 2004 and 2011 that had “Facebook” listed as one of the applicants, aggregating them into simple “families” of patents to avoid repetitions. This returned a total of 429 documents, which were exported into a spreadsheet. After deleting patents that were merely acquired but not submitted by Facebook employees and discarding those that did not directly pertain to social media functionalities, 330 documents remained. To ensure that the inventions would be as representative of the organization’s thinking as possible, we focused on 133 documents whose authors were or went on to become leaders in the company – founders, executives, vice-presidents, heads, and leads. This identification was carried out by manually searching for public information regarding the inventors’ names on professional platforms such as LinkedIn. A few of the techniques they describe – the news feed and platformisation systems – have been exhaustively studied before (Bucher, 2018; Helmond, 2015); others (such as social advisement) have been largely ignored.

We analysed the 133 documents manually, focusing on two standard sections. An invention’s “background” provides a justification for its existence and, in so doing, allows readers to locate and consider the problems inventors are addressing. When writing this section, we tried to ascertain whether and how those stated problems relate to the social world – did the technology concern an inherent social issue, such as how to define and control what counts as objectionable content, or was it designed to optimise an explicitly commercial aspect of the platform, such as how to improve the efficacy of advertisements in stimulating user attention? More than classifying these differing types of problems, we considered the ways in which such goals became entangled, and the consequences of their relations for how Facebook’s foundational technologies exerted power.

The second, and much longer, section we examined was the patent’s “detailed description,” where inventors explain their creation: what it is, which parts and processes it is composed of, what these parts and processes do, and how they relate to each other. The “detailed description” tends to be heavy in legal jargon. But, particularly when discussing the examples of how the invention would be enacted in real life (its “embodiments”), it allows readers to identify and interrogate inventors’ design decisions – how they decided to tackle the problems identified in the “background” section. To be clear, inventors rarely discuss these decisions explicitly. Patents are overwhelmingly descriptive texts, invested in the objective work of explaining what the invention is. It is only when one remembers that no pre-ordained single solution exists to the problem stated in the “background” that it becomes possible to denaturalise each aspect of these descriptions and understand them as what they are: the result of specific design choices, which can be pinpointed and critiqued.

We examined whether and how these problems and choices relate to understandings of the actual social and, in particular, three macro categories: social ontology (what the social world is), social normativity (what the social world ought to be, or at least aspects of it), and practices (the meaning of human interaction). These categories, which are congruent with how Berger and Luckmann (1966) and Honneth (2014) understand the social world, offered us an analytical pathway to explain in more specific terms how the social appears – and does not appear – in the patents. Taken as a whole, they allowed us to consider the extent to which Facebook’s early designers understood the immense complexities of actual human life, how their inventions would affect these complexities, and whether these systems embodied, directly or not, the sort of recognition that can be expected from social designers, as explained in the previous section. In practice, we toggled between coding and annotating text directly from the documents and recording our insights in a spreadsheet.

While patents are arguably the richest form of publicly accessible document about the early Facebook’s technologies – and despite the fact that all inventions we examined concern types of systems that the company indeed implemented – working with these data entail certain limitations. First, these patents are not necessarily complete descriptions of all the concrete systems the company used in that period, much less the entire field of social media. Moreover, patents offer just one static and inherently bounded picture of the company’s technological development – a complex and dynamic process. Finally, our approach cannot provide a sociological study of the actual motivations of individual Facebook engineers. Instead, by analysing the inventions these individuals wrote and published, we were able to demonstrate the constitutive recklessness that results when engineers build socially consequential products with reference only to their banal technical features, not those social consequences.

## The Social, According to Facebook

Using representative excerpts of the patents^4^ we examined, this section explores three assumptions about the social world that underpinned Facebook’s foundational technologies. We name them ontological plasticity, probabilistic normativity, and simulated sociality.

### Ontological Plasticity

In engineering the social graph, Facebook takes a peculiar approach to social ontology, making it so plastic that it becomes, ultimately, meaningless. Rather than explicitly proposing or enacting competing definitions of social life’s building blocks, their technologies often treat these elements as ultimately devoid of any inherent significance and, as such, amenable to all forms of top-down manipulation. This is evident not in their approach to contentious social categories such as gender, race, and class – which are rarely discussed in the patents we examined – but in much more fundamental elements: actors, relations, and actions, and how together they make up Facebook’s social graph.

These might appear to be arcane subjects of merely technical interest. Yet, several early documents delve into them, especially those that explicitly discuss what Facebook was intended to be. Company leaders typically describe their emerging creation as a “social networking system” that maintained a co-productive relationship with the “social graph” – a term inspired by “network theory” (Narayanan et al., 2010: 1[p]). This “graph” is seen as the computational space where some human and non-human entities (nodes) and the connections between them (edges) exist. A node is “something that can act on and/or be acted upon by another node”; an edge, in turn, is that which “represents a particular kind of connection between the two nodes, which may result from an action that was performed by one of the nodes on the other node” (Lindsay & Himel, 2010: 4[p]).

This definition suggests how nodes and edges are always assumed to be interdependent: a node cannot exist on its own since it is defined by its capacity to be connected to other nodes, and an edge can only exist between two nodes. While the idea of action is not explicitly defined, it is pervasively implied as something generative of actors and relations: it is the possibility of interaction *between* entities that makes networked reality representable in the first place. Indeed, the commercial “success of a social networking system” is defined as hinging on, ultimately, the company’s ability to “encourage users to interact” (Wang et al., 2010a: 1[p]).

More than interdependent, the entities of the social graph are also defined as interchangeable. Their meaning is always relational, with no hard conceptual boundaries between them. The exact same element can, for the purposes of the social graph, be several things at once (and even be discarded, i.e. cease existing) due not to users’ beliefs or wishes but because Facebook chooses to make and unmake them. Rasmussen et al. (2011: 1[p]) explain that, for instance, “messaging” (a user action) can be *or* create an “edge” between “two nodes”, but it may itself be “treated as a node”, in the same way that, when a user “tags” another user in a picture, this action (an edge in itself) may create connections “between each of the users” and the picture – itself “also a node”.

The looseness with which actual social categories and data objects of the social graph are associated is made transparent in a patent about “conceptual nodes”. These nodes could encompass “virtually anything” in the “real world” that users might be interested in, such as “a sport, a sports team, a genre of music, a musical composer, a hobby, a business (enterprise), an entity, a group, a third-party application, a celebrity, a person who is not a registered user, etc.” (Narayanan et al., 2010: 2[p]). In other words, every single action automatically becomes a data connection between nodes and a potential node in itself, even if users do not understand those actions as such. Thereby, all actions become *inter*actions. The very “network” concept has complete computational flexibility: it “may comprise people grouped according to *any type* of category [emphasis added]” (Bosworth & Cox, 2006: 4[p]).

Patents do not explicitly discuss why the data points that populate the social graph should not be categorised in any fixed way. But this is hardly a neutral sociological decision. In treating these data structures as semantically empty, Facebook maximises their utility for the purposes of value extraction. For instance, the data associated with a user’s personal characteristics (their location, for instance) might not be ordinarily understood as a relation, from the users’ perspective. Yet, in analytically treating it as such (say, as an “edge” between the user and a given city), the company enhances its capacity to infer and, then, materially engineer other user-user relations (e.g. between the user and local advertisers) that directly serve its business goals.

The “data structures” of the social graph must of course reflect existing elements of the “real world” in some minimal way if they are to shape “the social networking user experience” (O’Neill et al., 2011: 2[p]). But Facebook will interpret – and act upon – these structures regardless of what users think of them. Above all, it is the social graph’s own computational ontology, with its associated material properties, that enables Facebook to create a domain on which it can act almost entirely without reference to social meaning.

### Probabilistic Normativity

The engineering of the social graph involved not only creating “data structures” (potential nodes or edges) but also constantly analysing these elements so as to classify and organise them according to categories useful to Facebook’s business goals (growing their user base, incentivising people to spend as much time as possible on their platform). For these categorisations to be effective, they had to respond not just to purely computational problems but in some way also to needs in the actual social world. The patents we analysed focus on three such needs: relevance (essential to organising content in, e.g. news feeds that made interaction more likely), realness (key to avoiding fraud and expanding the usefulness of Facebook for other services), and objectionability (the basis of early content moderation systems).

Remarkably, instead of translating fixed normative views of these three ideas into computational code, Facebook’s foundational technologies resort to probabilistic techniques. To understand what we mean, consider first the definition of nodes and edges as “relevant” for the purposes of the social graph. While users can, in some patents, participate in the definition of what counts as important for them by prioritising or filtering content, relevance categorisation is generally discussed as automatic and based on other data points. One of the first news feed patents describes relevance as a function of the frequency and recency “of user interaction with other users and objects” (Zuckerberg et al., 2006: 3[p]), since these are what are most likely to generate engagement. The establishment of numerical thresholds, coefficients, and scores is critical for the treatment of these data (e.g. Tseng, 2010[p]) – but in a way that is explicitly tweaked to benefit Facebook. For instance, in a patent about a system aimed at recommending probabilistically “relevant” friends to users, Wang et al. (2010b: 2[p]) explain that this technology should not focus on “users with many connections”. Doing so would “lead to a sub-optimal result *for the social networking system* [added emphasis], since an additional friend for a user with many friends is less valuable [that is, commercially] than an additional friend for a user with relatively few friends” (Wang et al., 2010b: 2[p]). Such actions, which fed into the platform’s recommendations to users, were optimised not for what users wanted to be understood, but for what the company needed to happen.

Determining what counts as a “real” user, often based on behavioural evaluations, obeys a similar logic. A “valid” account was likely “to engage in safe or quality activities” that are aligned with Facebook’s focus on engagement, such as “establishing connections with other users; sending messages to other users; posting stories, pictures, or links on profiles; participating in online discussions; commenting on other users’ posts; and joining groups, events, or communities” (Underwood et al., 2010: 5[p]). And, as with many others, these operations depend on creating quantitative “thresholds” to define the probability of fraud as high enough to categorise an account as “fake”. These definitions can involve subjective judgments on which a parameter has to be applied: “the minimum fraud probability threshold *may* [emphasis added] be set by the social networking system based on a global policy, *or* the threshold *may* [emphases added] be set based on other factors.. such as demographics.. [and the] celebrity status” of the potentially fake user (Rubinstein & Singh, 2011: 6[p]). That is, while Facebook truly seeks to understand whether fraud occurs, these determinations are neither objective nor binding – they remain open enough to become subordinate to the platform’s ad hoc interests, if needed.

Third, multiple inventions explain how to define which node, from users to content, is “suspicious”, “malicious”, or somehow “objectionable”. Users can report these nodes (see e.g. Rubinstein et al., 2011). But from Facebook’s perspective, an approach that relies exclusively “on user feedback.. may be slow and sporadic”, leading users to “stop using the social network” due to the tarnishing of the platform’s image (Bosworth et al., 2007: 1[p]). Moreover, users’ reports are influenced by their “bias and personal preferences” (Kelmenson & Willner, 2010: 1[p]). Once again, behavioural data and probabilistic operations play a central role in enacting flexible automated mechanisms that, instead of enforcing any fixed moral view of objectionability, can moderate content in ways that still incentivize engagement. One invention explains how, in order to curtail “objectionable behaviour”, Facebook had to determine “whether a policy threshold for that type of action is exceeded”, and what would happen “when the combination of the current action and prior actions by the same user exceeds a predetermined level of activity” within a certain period of time (Bosworth et al., 2007: 1[p]). Yet such “policy” could differentiate between “activities” and “interactions” that are more or less desirable for Facebook: “[t]hresholds may be higher for actions that affect other users within the same group than for actions that affect users outside the group”, a distinction that was important to “promote intra-group activity” (Bosworth et al., 2007: 4[p]).

Probability enabled the platform to synthesise its incessant streams of data into apparently simple – and profitable – mechanisms that would make quasi-moral decisions on behalf of engineers without directly implicating these engineers in those decisions. Behind these operations is a view that platform norms should remain under the control and serve the interests of the company – instead of the social world they originally emerged from. The result is that moral accountability all but disappears, turning normative issues (as they appear in the actual social world) into a distant background. Such distancing is, Klein (2014: 270) notes, a key feature of how colonial extraction proceeds without embarrassment to those it benefits.

### Simulated Sociality

As is clear from the examples above, producing interaction – between users but also between users and things, place, ideas and indeed anything that could become a “data structure” – is an objective most of Facebook’s foundational technologies share. This computed form of sociality (Alaimo & Kallinikos, 2017) would guarantee the constant expansion of the social graph, making it ever more complete, granular, and thus, profitable. But these associations are never ends in themselves. The engineering of the social graph hinged on the assumption that, on Facebook, human interaction could perform functions and follow goals that benefited Facebook’s business model but which users could hardly understand when engaging with the platform’s interface.

This view of sociality as available for manipulation via a simulated double (the social graph) is present in *all* inventions, which either detail or take for granted that Facebook could lure users into thinking that they were acting socially while unknowingly participating in Facebook’s attempt to record, in digital data form, all elements and aspects of users’ “real life” – what one document calls “social mapping” (Zuckerberg & Sittig, 2006: 1[p]). A patent from 2011, for instance, describes a system that, under the guise of allowing users to upload videos or photographs, uses facial recognition to identify other people in the images. When explaining possible implementations, the document says that the system can automatically recognise actors appearing in a TV clip a user uploaded, infer which television program a user watches and then deliver targeted advertisement (Papakipos & Papakipos, 2011[p]). That is, people might think they are communicating about a personal event or posting about their favourite show but, from Facebook’s view, this was mainly about improving what they knew about users in a monetisable manner – not enabling self-expression. Another technology allows users to compliment others based on experiences listed on their profiles, such as work or education (Deng et al., 2010[p]). But again enabling mutual recognition is not the most important objective of the invention. In fact, Facebook incentivises these compliments to “formulate a reputation for the user in the domain associated with the complimented experience, and/or to establish a reputation ranking among users for experiences in the domain” (Deng et al., 2010: 7[p]). Once these ratings and rankings are in place, they can be used to help professional and commercial users (e.g. recruiters) find reputable professionals or help define the content users can get in their feeds (Deng et al., 2010: 10–11[p]) – including ads. One patent is entirely designed to help “reactivate” a “dormant” user by initiating “an *intervention scheme* [emphasis added] where the user is recommended to take actions that induce interactions with the low-activity users” (Wang et al., 2010a: 2[p]). Instead of merely engaging with a friend that has been a little silent, users are incentivized, unwittingly, to contribute to Facebook’s project of prodding users to engage with their products and produce data through interaction.

The most basic type of behaviour designed by Facebook’s social graph involved the voluntary provision of information about themselves, others, and their world. To users, these were, perhaps, playful identification acts; to Facebook, they had an engineering purpose, allowing the creation of new nodes and edges with the social graph. While “[a]ny type of data may comprise the biographical data” (Zuckerberg & Sittig, 2006: 4[p]), these statements were mostly demographic and about “interests” and “views”. Another form of self-declaration emerges in what today would be called users’ own “data work” (Miceli & Posada, 2022): the annotation of already existing ‘data structures’ representing the “real world” to generate new nodes or edges. Multiple patents explain how important it was for Facebook to *allow* (in fact, incentivize) users to “tag” or “check in” themselves, other users, non-users, companies, and virtually anything else on posted pictures and locations. Non-users could reject a tag – but for that, they would have to first create an account (Zuckerberg et al., 2010[p]), another benefit potentially to Facebook. Tags also helped Facebook evaluate existing connections according to their “realness” from the perspective of a social graph. After all, if “two users.. checked-in to a restaurant at the same time” or were “tagged in the same photo”, there was a “high likelihood” that they knew each other “in the real world” (Rubinstein et al., 2010: 18–19[p]). Facebook could also automatically create edges and nodes based on users’ voluntarily given information – without their knowledge. Narayanan et al. (2010: 23[p]) illustrate this sort of system with the example of someone who, by merely typing “I love climbing in northern California” on their profile, triggers a “backend” process in which the platform would either automatically associate this person with a “node” about climbing in Northern California or, in case such a node did not exist, engineer a new node right away.

Even technologies that might appear benevolent or beneficial to users entailed hidden goals and meanings. Defining a profile as “real” appears as an opportunity for the platform to secretly collect GPS and biometric data, such as facial and voice data (Gandhi & Papakipos, 2011: 5[p]) or behavioural data from external websites (Underwood et al., 2010: 8[p]). Another technology promised to protect users’ privacy – an aspect which is embedded in many of the documents we examined. In fact, the earliest patent we collected concerns precisely “systems and methods for dynamically generating a privacy summary”, i.e. selecting audiences for one’s posts (Zuckerberg & Kelly, 2006: 1[p]). In leading users into thinking that a post’s differential visibility on the platform somehow equates to a type of privacy, the platform endowed people with a false sense of control that was conducive to increasing usage – but not to protecting their privacy, necessarily.

## Engineering the Social Blind

This article has set out to explore what we have termed the indeterminacy of social media – that is, the chasm between platforms’ apparently trivial goal of designing profitable social graphs and the epoch-making consequences that these products have generated in the actual social world. In conclusion we argue that driving this indeterminacy was not social values, let alone a worked-out view of social engineering, but instead banal practices of engineering that were entirely reckless about their actual social consequences.

As demonstrated above, Facebook’s foundational technologies appear to assume that actual social ontology, norms, and practices can be freely renamed and reinterpreted, produced by decontextualised quantitative processes, and lead to reconfigurations and combinations whose multiple, uneven significance is all but invisible to users. We argue that, together, these understandings compose a broader assumption of human life as a *terra nullius*, a domain which can be simulated and then operated upon without any attention to inherent social meaning or value, because, it was assumed, there were no relevant social actors to be taken into account. Our use of this term is inspired by Julie E. Cohen. She argues that platforms constructed a "biopolitical public domain" that "*subordinates* [emphasis added] considerations of human well-being and human self-determination to the priorities and values of powerful economic actors" (Cohen, 2019: 73), and defines personal data as “a *terra nullius* for enterprising data developers, an unexplored frontier to be staked out, mapped, and colonized” (Cohen, 2019: 51; compare Couldry & Mejias, 2019: 9; Mejias & Couldry, 2024: 37). What the patents we discussed suggest goes arguably even further. Not only "personal data", but the wider social world, was seen as no-one’s territory. Underlying this possibility was the executive power that Wendy Chun noted is basic to all computer codes: a banal ‘autonomy’ that is intrinsic to computer code which "exists first and foremost as commands issued to a machine" (Chun, 2011: 22; cf. Galloway, 2004). But combine this with the overriding goal of maximizing engagement by nudging activities (of real social actors) related to the social graph, and code starts working *on* actors in the real social world, even in the absence of any vision *of* the social world.

The assumptions we identified in the patents do not suggest social planning of any sort; they are focussed entirely on the manipulation of the social graph, as mediated by the entities counted within it, and the company’s control over it, with the resulting ability to monetise life. The effectively ‘synthetic’ world on which Facebook engineered sought to act had stringent operational rules – all must be captured so as to be connected – but no overarching moral norms or guidelines. This might be said, perhaps, of all analysed patents which, after all, are designed only to give economic protection to the parameters of an invention, but it is particularly ironic in systems which at times explicitly aimed to make morally-laden definitions such as what counted as ‘real’, ‘objectionable’, or ‘relevant’ (Zuckerberg et al., 2006; Underwood et al., 2010; Bosworth et al., 2007).

Facebook’s vision of the social as a void terrain enables a form of *socially blind engineering*: the systematic design of a world comprised of components which lie actual social referents, but which, instead of having the social in view, is marked by a wilful disregard for the social consequences of the large-scale power invested in platform engineers. To borrow a phrase from Pierre Bourdieu’s *Outline of a Theory of Practice* (1977/2013: 77), "everything takes place as if" something like social engineering has gone on: there has been a structured reshaping, indeed a reconfiguration, of social life whose key features can, in precise ways, be linked to features of social media platforms. Bourdieu however used that phrase ("everything takes place as if") to describe large-scale social processes that are immanent in social life when we look across the overlapping intentions of countless human actors. But that is not exactly the case here: the appearance of social engineering has emerged from the actions of billions of platform users *combined with* the highly concentrated and deeply asymmetrical power of social media corporations and their very particular software designs.

Viewing the social as an empty terrain whose mechanics could be freely re-engineered suggests that designers failed to comply with their most basic obligation – to properly recognize users as both able to and deserving of social freedom, as defined above. This failure seems to have taken a rather particular form. It is not that designers reified users and did not think of them as humans subjects, what Honneth (2008) would have called antecedent misrecognition. The documents we examined demonstrate that the creation of Facebook, as a technology, is entirely premised on the idea that users, as other humans, want to incessantly interact. It does not appear to be the case, either, that inventors failed to understand that users seek recognition from others. This aspect was acknowledged by Sean Parker, the company’s first president. Speaking after the first election of Donald Trump in 2016, when the company was under extreme scrutiny, Parker said that the platform was designed to commercially exploit a “vulnerability in human psychology”: “self-validation” (Solon, 2017, para. 1). His statement confirms what is strongly implied in the patents: that Facebook acted on and amplified our unavoidable, fierce desire to be heard and socially recognised for reasons that are not primarily social or moral but selfishly economic. To put it another way, they instrumentalised users’ fundamentally moral process of *trying* to achieve recognition by enabling ever-more elaborate forms of expression and reactions that could appears as ‘like’ recognition, while, at the same time, ignoring whether the result might, in fact, be to enhance actual recognition or respect. Far from creating a “neutral” space, as some of its creators still claim to have done, Facebook determined that all preexisting normative dimensions of the social would have to compete for recognition in a space ruled by the company’s own mechanistic principles of capture and connection. Facebook’s fundamental misrecognition of the social world stems not from ignoring that human individual recognise each other in morally relevant ways, but from acting on processes of recognition in ways that, instead of being moral, were focussed on one goal only : generating activity, data and, eventually, profit.

In knowingly deciding not to think about its own social power, the platform put in motion a process that was neither accidental nor intentional but banal, in the sense that bureaucracy is banal. Banality is essential to make sense of Facebook’s social blindness. It is the self-serving status of the social graph – its absence of anything like principles of *social* design – that generates the moral hazard of commercial social platforms for the actual social world. This banality (its standing within a domain of pure engineering) is based in a merely operational authority whose closest analogy is bureaucratic power (Fourcade & Healy, 2017: 10–11). Commercial social media’s recklessness, indeed amorality, derives then not from a substantive but misguided social intention, but from the absence of any such intention, beyond the pursuit of whatever functional properties aided the social graph. It is the banal *absence* of social intention in Facebook’s engineering (its seclusion within a domain which prima facie, unlike state bureaucracy, had no relevance to social policy or population management whatsoever) that perhaps prevented regulators seeing the risk posed by platforms operating on the social world as if its actors did not need to be taken into account.

While this article has focussed on, and is empirically limited to, the early version of Facebook, we offer our critique as potentially an exemplary case with wider implications. In fact, all organizations that design data territories (Mejias & Couldry, 2024) without a clear view of the sorts of social change that their code might engender risk engineering the social blind. Of course, any assessment of the generalizability of our conclusions must involve new and detailed empirical analysis of those other platforms’ functions (including any relevant patents). Yet there is public evidence of how platforms’ wilful disregard for their own power over the social world may remain an alluring paradigm across data-extracting companies. Meta’s ideological swing towards the far-right after the election of Donald Trump in 2024, and the subsequent degrading of its fact-checking in (at least) the US, demonstrated an utter lack of concern for their billions of users’ right to be well-informed (Levitz, 2025). Similarly, the extraordinarily fast and widespread deployment of generative AI in multiple spheres by essentially all Big Tech firms suggests a fundamental recklessness that, as Napoli and Adi (2025) explain, echoes the conditions in which social media was invented. These developments only underline the importance of carefully identifying, as we have attempted in this article, the roots of the tech sector’s irresponsibility: designing systemic social infrastructures without attention to their constitutive implications for how social life is actually lived.

## Data Availability

The dataset on which this article is based is available upon request.

## Footnotes

1 While digital platforms are not limited to social media, this article uses these two terms interchangeably.

2 If the social graph is defined as the datafication of social relations and the prioritization of these relations in determining content visibility, it is no longer the dominant model. All major platforms have recently, in some form, adopted TikTok’s commercially successful recommendation approach, where the likelihood of user engagement with a piece of content outweighs the importance of who posted it. However, if the social graph is more broadly defined as the data representation of users, their actions, and their relationships – regardless of which aspect is prioritized in the feed – it remains the computational backbone of all social media.

3 Wynn-Williams (2025) provides extensive evidence of this.

4 To facilitate the identification of patents in the in-text citations, we added the expression [p]; years correspond to their filing date.
